# Propagule pressure and an invasion syndrome determine invasion success in a plant community model

**DOI:** 10.1002/ece3.8348

**Published:** 2021-11-13

**Authors:** Daniel Vedder, Ludwig Leidinger, Juliano Sarmento Cabral

**Affiliations:** ^1^ Ecosystem Modeling Group Center for Computational and Theoretical Biology University of Würzburg Würzburg Germany

**Keywords:** community trait analysis, individual‐based modelling, island plant communities, propagule pressure, species invasions

## Abstract

The success of species invasions depends on multiple factors, including propagule pressure, disturbance, productivity, and the traits of native and non‐native species. While the importance of many of these determinants has already been investigated in relative isolation, they are rarely studied in combination. Here, we address this shortcoming by exploring the effect of the above‐listed factors on the success of invasions using an individual‐based mechanistic model. This approach enables us to explicitly control environmental factors (temperature as surrogate for productivity, disturbance, and propagule pressure) as well as to monitor whole‐community trait distributions of environmental adaptation, mass, and dispersal abilities. We simulated introductions of plant individuals to an oceanic island to assess which factors and species traits contribute to invasion success. We found that the most influential factors were higher propagule pressure and a particular set of traits. This invasion trait syndrome was characterized by a relative similarity in functional traits of invasive to native species, while invasive species had on average higher environmental adaptation, higher body mass, and increased dispersal distances, that is, had greater competitive and dispersive abilities. Our results highlight the importance in management practice of reducing the import of alien species, especially those that display this trait syndrome and come from similar habitats as those being managed.

## Graphical abstract

We use an individual‐based model to study environmental factors and traits affecting species invasion in island plant communities. We find that propagule pressure is the most important invasion success factor. Also, invasive species display a trait syndrome that is more highly adapted and dispersive than the native community.

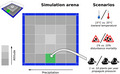

## 1 INTRODUCTION

Species invasions are highly complex phenomena, influenced by several interacting factors, such as species traits, disturbance, or evolutionary history (Enders et al., 2020; Theoharides & Dukes, 2007). Gaining an understanding of these factors is necessary to understand the whole invasion process (Fleming & Dibble, 2015) and establish effective countermeasures (Novoa et al., 2020). Yet, the relative importance of various factors is difficult to derive from studies focusing only on single invasion events (Catford et al., 2009). Considering the impending global change scenarios and increased rate of biotic exchange, however, generalizable findings about biological invasions are still urgently needed (van Kleunen et al., 2015).

In the last two decades, a number of factors have been identified that contribute to the success of species invasions. A prominent role falls to the number of introduced organisms, known as propagule pressure, as it ensures minimal viable population sizes (Carr et al., 2019; Lockwood et al., 2005). Abiotic factors such as enhanced productivity (i.e., rates of biomass production) and increased disturbance have also been suggested to facilitate invasions in some circumstances (Driscoll, 2017; Huston, 2004). Beyond these, species traits may also determine invasiveness, especially in the longer term (Kempel et al., 2013). Arguably the most obvious of these traits is sufficient pre‐adaptation to the abiotic environmental conditions of the invaded habitats (Carboni et al., 2016). Additionally, invasive species need to be able to compete with resident species to establish (Alzate et al., 2020). Lastly, increased dispersal abilities and broad environmental niche preferences, that is, generalism, will enable alien species to spread (Irl et al., 2021). All these invasion factors may vary in their level of expression, depending on the system and taxa.

It has been demonstrated that the combination of both environmental factors and species traits has considerable effects on the success of invasions (e.g., Küster et al., 2010; Thuiller et al., 2006). To address such interactions, Catford et al. (2009) proposed an experimental design that varies propagule pressure, species composition, and abiotic conditions in a full‐factorial setup to assess their relative importance for invasion success. An experimental approach like this will be necessary to arrive at a generalized understanding of the invasion process. However, few studies consider such a broad spectrum of factors.

For generalizing invasion processes, islands can be useful model systems. Firstly, islands are highly susceptible to invasion‐related degradation (Nogué et al., 2021). For example, invasive predators have caused multiple species extinctions on islands (Doherty et al., 2016), and there have been observed cases of complete “invasional meltdown” after native keystone species were displaced (O'Dowd et al., 2003). Secondly, their small size, isolation, and comparatively simple ecological dynamics mean that islands are popular study systems in ecology in general (Patiño et al., 2017). Observational and correlative studies are becoming increasingly feasible as more biodiversity data are becoming available (e.g., Irl et al., 2021; van Kleunen et al., 2019). Still, collating and analyzing such data from a variety of sources is complicated (Isaac et al., 2019). Also, invasion experiments continue to be difficult to conduct at large spatial and temporal scales even on islands, due to practical challenges and the ethical risk of experimentally introduced organisms escaping (cf. Russell et al., 2005).

As an alternative, mechanistic models offer a powerful approach to supplement field studies. Such models have previously been used in invasion biology, although often in the context of specific invaded sites (e.g., Davis et al., 2019; Iannone et al., 2014). However, they also hold great promise for exploring fundamental processes within a more generalized setting (Cabral et al., 2017; Grimm & Railsback, 2005; Leidinger & Cabral, 2017) and are very useful for gaining a mechanistic understanding of complex ecological patterns (Grimm & Railsback, 2011). The fact that mechanistic models allow both complete control over all environmental variables and complete knowledge of every organism's traits makes them an ideal tool to help us better understand the intricacies of the invasion process.

Here, we therefore used a recently developed individual‐based mechanistic model of island plant communities (Leidinger et al., 2021) to investigate the factors of invasion success. The model explicitly simulates genotypes and phenotypes of all individual plants in a randomly generated species assemblage, as well as their interactions with each other and their environment. After allowing native species communities to develop on the simulated islands, we introduced non‐native species and observed under which conditions these were likely to undergo landscape spread, that is, become invasive.

We set up our experiment as proposed by Catford et al. (2009), which allowed us to ask the following questions: (1) “How important is propagule pressure?”, (2) “What effect do productivity and disturbance have?”, and (3) “How does the trait composition of invasive species differ from native species, or from alien species that fail to become invasive?”. We find the most influential factors of invasion success to be propagule pressure, as well as a trait syndrome comprising strong dispersal abilities, higher biomass, and good environmental adaptation.

## 2 METHODS

### 2.1 The model

**FIGURE 1:**
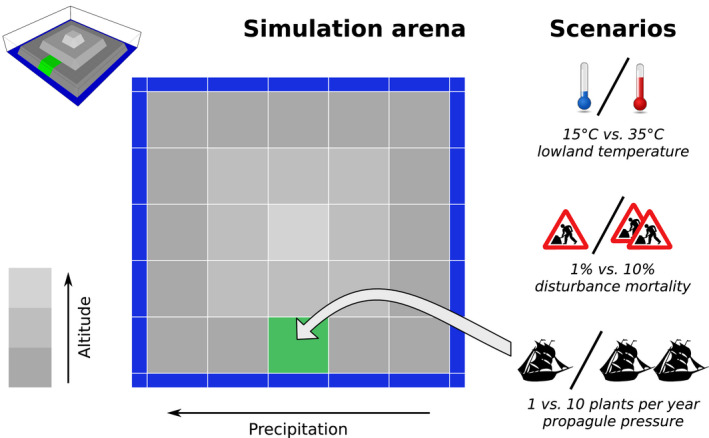
Schematic representation of the simulated island. Grid cells are one‐hectare habitat patches. Temperature decreased with altitude, giving a radial gradient with a step size of 2°C per unit of height (gray scale). A second gradient of an abstract environmental resource (termed “precipitation” for simplicity) was applied longitudinally. The green grid cell denotes the point of entry for alien species (the location of this was identical in all simulations). Pictograms show the three factors that were varied in the experimental setup, namely temperature, disturbance, and propagule pressure. The small inset depicts a three‐dimensional rendering of the island for illustrative purposes

We extended the genetically explicit metacommunity model (GeMM) of Leidinger et al. (2021) to simulate species invasions to plant communities on a virtual oceanic island (Figure 1). The island consisted of a 5 × 5 grid depicting a radial elevation (and corresponding temperature) gradient. Additionally, there was a linear precipitation gradient, which is typical for many oceanic islands that lie in the path of prevailing winds. We call this second gradient “precipitation” for simplicity, although it could also be interpreted as any other environmental characteristic that exhibits a gradient. Each grid cell was assumed to be one hectare in size, with a biomass carrying capacity of two tonnes. Each cell could hold its own community, comprised of individual plants belonging to one or more species. In the GeMM, species are abstract entities and not meant to be direct representations of any one actually existing biological species. However, as individual body sizes in the model could range between 150 g and 1.2 tonnes, our system can be thought of as simulating a grassland/shrubland ecosystem (cf. Deshmukh, 1984). Due to the level of genetic and ecological detail at which each individual plant is modeled, GeMM has high computational demands (with one simulation run in this experiment requiring about 24 h of computation time). Island geometry and the experiment's spatial and temporal extent were therefore chosen to ensure computational feasibility, as well as to provide different environmental combinations and to enable sufficient coexistence of native species.

In the model, each individual plant has a genome consisting of multiple genes that code for a set of traits, which in combination determine the plant's phenotype and were used as parameters of biological functions (Table 1). These traits encompass environmental optima and tolerances to temperature and precipitation conditions, seed size, reproductive (adult) size, and mean and shape parameters of a logistic dispersal kernel (Bullock et al., 2017; Lin et al., 2021). Reproduction takes place sexually between adult members of the same species and includes genetic recombination. Self‐pollination is not permitted; mutation is possible, but was turned off for this study to reduce evolutionary confounding effects. For performance reasons, we restricted pollen dispersal to the local grid cell; that is, plants do not reproduce with plants in other patches. (Previous model analyses by Leidinger et al., 2021, have shown this restriction to have little effect on population dynamics.) Gene flow is provided by seed dispersal, as juvenile plants disperse away in a random direction from the mother plant. Seeds establish and grow if they land in a patch whose environment (temperature and precipitation) they are sufficiently adapted to, or die if the patch is unsuitable or they disperse beyond the borders of the simulated island.

**TABLE 1:** Ecological and life‐history processes included in the invasion model, in the order of execution

Process	Details
Survival	Density‐independent mortality relative to an individual plant's temperature adaptation.
Growth	Plants increase in mass until they reach reproductive size.
Competition	If the total biomass in a grid cell exceeds its carrying capacity, compete pairs of plants and remove the one with the lower precipitation adaptation (density‐dependent mortality).
Reproduction	Sexual reproduction including recombination of the parents’ genomes by meiosis, produces multiple seeds.
Disturbance	Species‐ and density‐independent mortality of a given percentage of plants in each grid cell.
Transport	Introduction of aliens (i.e., plants from the alien species pool) into the point of entry.
Dispersal	Dispersal of seeds produced during reproduction, the distance is calculated with a dispersal kernel.

The probabilities for growth, fecundity (seed numbers), and density‐independent mortality were determined using the Metabolic Theory of Ecology (MTE; Brown et al., 2004, which links life‐history rates to metabolic processes dependent on body mass and the local temperature. Thus, large organisms have a greater number of offspring and a longer life expectancy than small organisms, and all process rates are increased by increasing temperatures (see Appendices S1 and S2). Density‐independent mortality was additionally linked to each plant's temperature adaptation, a value calculated from the plant's temperature optimum and tolerance traits in reference to the local patch temperature. We modeled competition for space (i.e., density‐dependent mortality) when the patch carrying capacity was exceeded by competing random pairs of plants and removing the one with the lower precipitation adaptation (a value calculated analogously to temperature adaptation).

At the start of the simulation, the island was initialized with randomly generated species. Species’ initial population sizes were dependent on their body sizes, so the number of species that could fit into the island carrying capacity varied as different simulation runs generated species of different sizes. To introduce standing variation, each individual plant's traits were slightly varied using a normal distribution centered on the species’ mean trait values. This initial island community was then allowed to co‐evolve over 500 years, which gave it time to reach quasi‐equilibrium with respect to species richness and population sizes. Depending on the simulation run, native species numbers after this “burn‐in period” ranged between 1 and 15, with a mean of six (Figure S2).

To act as a reservoir of alien species, a global species pool was generated consisting of an additional 100 species. After the burn‐in period, a number of plant individuals (depending on the scenario, but constant within each simulation) was drawn from this species pool every year and introduced to a specified grid cell on the island (“point of entry”; Figure 1). The species identity of each introduced plant individual was chosen independently, so if multiple plants were introduced in a given year, these could include multiple plants of the same species. To further mimic potential effects of the onset of any human activity on this small island (e.g., trampling, direct extraction, livestock grazing), disturbances also started after the burn‐in period, consisting of a given percentage of individuals being randomly removed from each grid cell every year, in addition to the previously mentioned causes of mortality. The model was allowed to run for a total of 1500 years.

For the choice of parameter values, etc., the reader is referred to the full model description in the ODD format (Grimm et al., 2010), found in Appendices S1 and S2. The source code for the model was written in Julia (Bezanson et al., 2017) and is available at https://github.com/CCTB‐Ecomods/gemm, along with its documentation.

### 2.2 Experimental design

We varied the three factors propagule pressure, productivity, and disturbance in a full‐factorial design across two levels of each factor to give a total of eight scenarios (cf. Catford et al., 2009; Figure 1). 60 replicates of each scenario were run, resulting in 480 simulations.

We used temperature as a proxy for productivity, setting the base (lowland) temperature to either 15°C or 35°C. Due to our use of the MTE, higher surrounding temperatures lead to an increase in growth and reproduction rates, causing overall higher rates of biomass production. Propagule pressure (1 or 10 individuals per year) and disturbance (1% or 10% mortality per year) were explicitly implemented in the model, as described above.

### 2.3 Data recording and analysis

Every 50 years, the model recorded a log file with the median and variance of each population's trait values. For our analysis, we concentrated on the state of the simulations at the end of the experiment. All data analyses were carried out in R using tidyverse and ggplot2 for analysis and visualization (R Core Team, 2017; Wickham et al., 2019).

**FIGURE 2:**
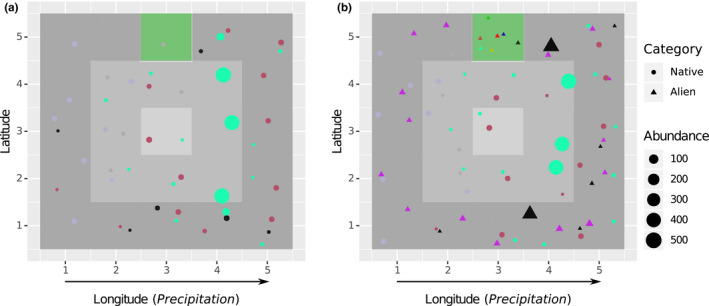
(a) Island map after the burn‐in period (t=500a) and (b) at the end of the simulation (t=1500a) for one example simulation run. Each marker represents one population; colors signify species. Natives are circles and aliens triangles. Population markers are jittered within their grid cell to avoid visual overlap in the figure. The green grid cell is the point of entry, and gray scale denotes temperature (cf. Figure 1)

To quantify the effect of the varying factors on the success of species invasions, we categorized species as native, alien, or invasive. Native species were species from the original island community that were still extant at the end of the experiment. Alien species were those that had been introduced to the island from the global species pool, but failed to spread beyond the point of entry. Lastly, invasive species were those that had been introduced and had established at least one population outside of the point of entry. (We acknowledge that there are different usages of the term “invasive.” As we do not investigate the impact that a non‐native species has on the native ecosystem, we follow Ricciardi & Cohen, 2007 and restrict our definition to species that have undergone landscape spread.) During each run, we generated island maps at regular intervals, showing size, location, and species of all populations (e.g., Figure 2).

To investigate the effects of propagule pressure, productivity, and disturbance (Questions 1 and 2), we identified all species that became invasive across all simulation runs and summed these up by scenario. To compare natives, aliens, and invasives by their traits (Question 3), we calculated their overall environmental adaptation, compared individual traits via linear mixed models and simultaneous max‐t tests using Tukey contrasts (from now on: “Herberich tests”), and additionally contrasted the trait space of natives and invasives using a PCA.

The overall environmental adaptation $A_{\text{ind}}$ of plants was calculated as the product of their temperature adaptation and precipitation adaptation:

(1) $$A_{\text{ind}}=G\left(T_{\text{opt}},T_{\text{tol}},T_{\text{env}}\right)\times G\left(P_{\text{opt}},P_{\text{tol}},P_{\text{env}}\right)$$

where G(b,c,x) is the Gauss function at point *x* with mean *b* and a standard deviation of *c*; $T_{\text{opt}}$ and $P_{\text{opt}}$ are the plant's temperature and precipitation optimum value, $T_{\text{tol}}$ and $P_{\text{tol}}$ its temperature and precipitation tolerance, and $T_{\text{env}}$ and $P_{\text{env}}$ the actual environmental temperature and precipitation values in the local grid cell.

For individual trait comparisons between alien, invasive, and native species, we pooled population data per species category from the final simulation year in all scenarios. The traits we were interested in were mean dispersal distance, long‐distance dispersal, precipitation tolerance, temperature tolerance, adult biomass, and seed biomass. Since precipitation and temperature optima traits were primarily influenced by geography and the particular temperature scenarios, we omitted them from our analysis of the pooled data. Furthermore, we log(x+1)‐transformed all trait and adaptation values to improve normality, because the original distributions were left‐skewed and contained values <1.

We then performed linear mixed models using the R packages lme4, lmerTest, and multcomp (Bates et al., 2015; Herberich et al., 2010; Kuznetsova et al., 2017). For this, we used the particular trait value as response, species category as fixed effect, and the specific simulation run as random effect. We report degrees of freedom and *p*‐values (calculated via Satterthwaite approximation method with the lmerTest R package) to facilitate comparison between species categories. However, we note that caution must be taken in interpreting *p*‐values for simulated results, as the significance can be guaranteed by increasing replicate number, and the fundamental assumption of comparing a sample to a “true mean” is invalid (White et al., 2014). Therefore, we also provide R^2^ to provide an estimation for the variance explained. To supplement these linear mixed models, we additionally performed Herberich tests for pair‐wise comparisons of species category (Herberich et al., 2010). Herberich tests do not account for random effects, but can deal with unbalanced, non‐normally distributed and heteroscedastic data, and are thus more robust than linear mixed models.

Lastly, in order to focus more closely on the differences between natives and invasives, we conducted a principal component analysis (PCA) on standardized trait medians of our data for these two categories. This allowed us to investigate general patterns of trait space by comparing the size and location of 95% confidence interval ellipses corresponding to the different species types.

## 3 RESULTS

**FIGURE 3:**
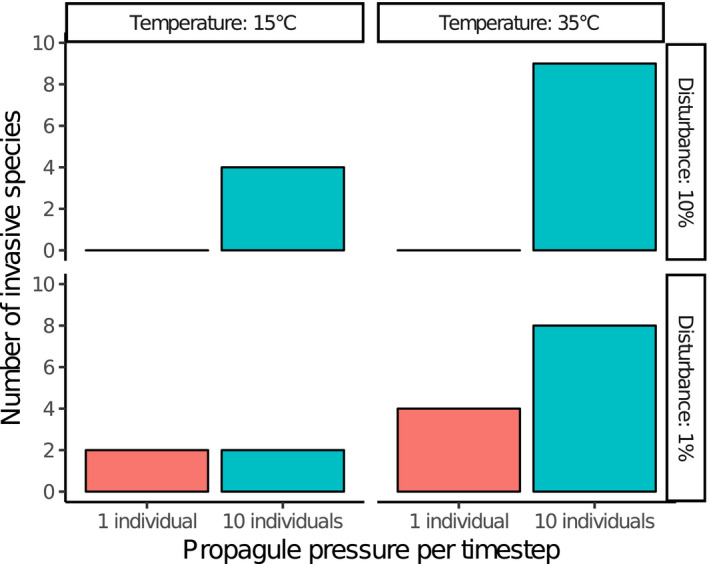
Cumulative number of invasive species observed in each of the eight scenarios (60 replicates per scenario)

A total of 28 species became invasive over all simulation runs. We found a strong link between propagule pressure and invasibility, with almost four times as many invasives occurring in high‐pressure compared with low‐pressure scenarios (Figure 3). Temperature also had a strong influence, with a twofold to threefold difference between levels. There was a slight positive relationship between disturbance and invasibility.

**FIGURE 4:**
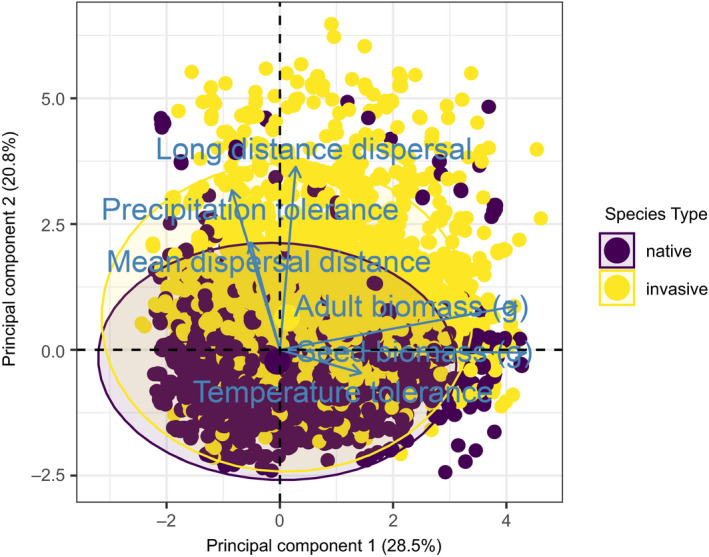
Principle component analysis (PCA) showing the distribution of population trait medians in the trait space. Each axis arrow represents one trait, and each marker is one population. All traits were log(x+1)‐transformed and normalized before PCA calculation. Natives are in purple and invasives in yellow. Ellipses represent 95% confidence envelopes

In terms of the total trait space, invasive populations exhibit a larger spread than natives (Figure 4). The center of the invasive populations' trait space is shifted along the second PCA dimension toward higher long‐distance dispersal, higher mean dispersal distance, and higher precipitation tolerance compared with native populations.

**FIGURE 5:**
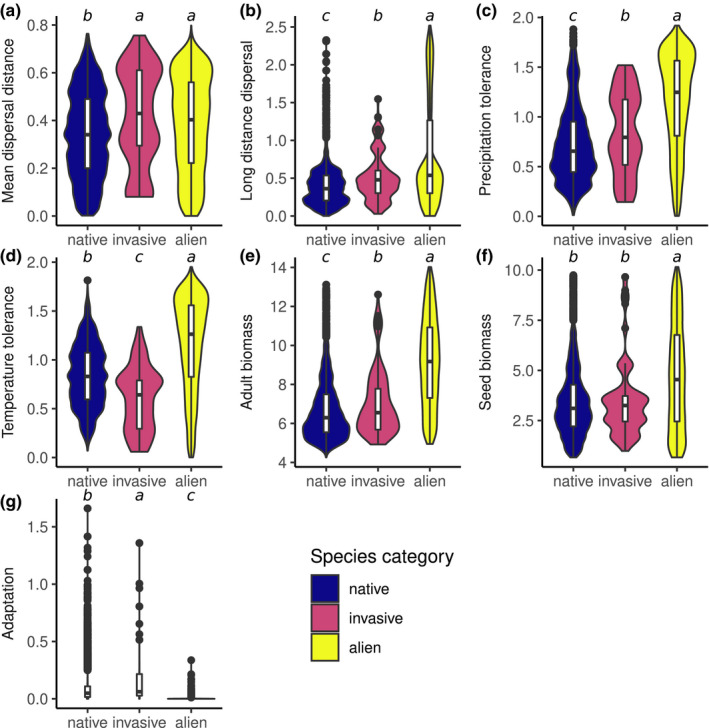
Distributions of single trait values. (a) Mean dispersal distance, (b) long‐distance dispersal, (c) precipitation tolerance, (d) temperature tolerance, (e) adult biomass in grams, (f) seed biomass grams, and (g) adaptation to local temperature and precipitation conditions. All values were log(x+1)‐transformed before visualization. Boxes show medians and interquartile range. Blue: populations of native species, red: populations of invasive species, yellow: populations of alien species. Letters designate pair‐wise significant differences (categories with different letters in a plot are significantly different to each other, and letters are allocated in order of descending means; Herberich et al., 2010)

The difference in total trait space is associated with specific differences of the particular traits between species categories. Specifically, mean dispersal distance (Figure 5a), long‐distance dispersal (Figure 5b), precipitation tolerance (Figure 5c), and adult biomass (Figure 5e) were all increased in aliens and invasives compared with natives. Temperature tolerance (Figure 5d) and seed biomasses (Figure 5f) were increased in aliens, but slightly decreased for invasives. For long‐distance dispersal, precipitation tolerance, temperature tolerance, and adult and seed biomass, invasives were closer to the mean of natives than aliens were. Invasives achieved the highest median adaptation values, although natives had higher maximal values. Alien adaptation values were very low (Figure 5g).

The results from the linear mixed models revealed what traits were most important to distinguish invasive species from native (Table 2). Whereas invasive species differed to native species most significantly in regard to mean dispersal distance and temperature tolerance, aliens generally were evidently different across all traits. The difference in long‐distance dispersal, precipitation tolerance, and adult biomass explained the most variance, followed by temperature tolerance, seed biomass, and mean dispersal distance.

**TABLE 2:** Results of linear mixed‐effects model fits comparing species’ traits values with species category (native, alien, or invasive; with native as reference) as fixed effect and replicate as random effect

Trait/model	*R* ^2^	Fixed effect	Estimate	Std. error	df	*t* Value	*p* Value
Mean dispersal distance	.206	(Intercept)	0.344	0.004	475	85.981	<.001
		Invasives	0.086	0.012	29,850	6.928	<.001
		Aliens	0.040	0.003	30,410	13.879	<.001
Long‐distance dispersal	.552	(Intercept)	0.469	0.014	444.7	34.200	<.001
		Invasives	0.037	0.022	30,370	1.731	.0835
		Aliens	0.325	0.005	30,160	64.904	<.001
Precipitation tolerance	.403	(Intercept)	0.771	0.011	473.2	68.498	<.001
		Invasives	0.015	0.025	30,450	0.578	.563
		Aliens	0.381	0.006	30,300	65.366	<.001
Temperature tolerance	.28	(Intercept)	0.820	0.007	473.8	110.04	<.001
		Invasives	−0.253	0.024	29,800	−10.74	<.001
		Aliens	0.342	0.005	30,410	62.32	<.001
Adult biomass	.408	(Intercept)	6.849	0.044	478.6	155.483	<.001
		Invasives	−0.104	0.110	30,370	−0.946	.344
		Aliens	2.277	0.026	30,340	88.751	<.001
Seed biomass	.243	(Intercept)	3.511	0.042	473.7	84.487	<.001
		Invasives	−0.202	0.127	29,920	−1.593	.111
		Aliens	1.194	0.030	30,400	40.305	<.001
Adaptation	.188	(Intercept)	0.086	0.002	477.5	41.743	<.001
		Invasives	0.074	0.008	28,546.6	9.425	<.001
		Aliens	−0.081	0.002	30,453.2	−44.23	<.001

Abbreviations: df, approximate degrees of freedom; *R* ^2^, conditional *R* ^2^; Std. error: standard error.

## 4 DISCUSSION

### 4.1 Propagule pressure

Concerning the importance of propagule pressure for the success of invasions, our findings positively answer our first study question, following the literature‐based expectations and mirroring ample evidence from empirical studies, including macroecological analyses (e.g., Carr et al., 2019; Seebens et al., 2018). Indeed, propagule pressure is well‐known as the leading driver of invasion success in the current literature (Cassey et al., 2018; Lockwood et al., 2005). This could be explained by Allee effects in introduced populations (Keitt et al., 2001; Taylor & Hastings, 2005). That means that only sufficiently large populations will grow fast enough to overcome adverse abiotic conditions and inter‐specific competition by native species. As described in Allee effects literature, this relation of population growth to density is typically hump‐shaped and will decrease again beyond a critical density (Courchamp et al., 1999; Stephens et al., 1999). Thus, we can assume that increasing propagule pressure will not increase invasion success indefinitely.

Indeed, Cassey et al. (2018) found a sigmoidal relationship between propagule pressure and establishment success. This was also reflected in additional post hoc experiments with our model. With propagule pressure increased to 100 individuals per year, we did not observe an increase in the number of successful invasions (Figure S1). This propagule saturation is likely caused by the filling of the island community's functional or niche space. Whereas early species introductions benefit from island communities that are frequently disharmonic (i.e., do not fill the available niche space; Whittaker & Fernández‐Palacios, 2007), these introductions also reduce the remaining niche space. Thus, later species introductions are less likely to be able to establish based on niche difference and have to rely on fitness advantage (MacDougall et al., 2009).

As regards propagule pressure, it is also important to point out that previous studies frequently conflated this with the related but separate concept of colonization pressure, that is, the number of species (rather than individuals) introduced per time (Lockwood et al., 2009). Although the two may interact and are difficult to differentiate in the field, they do in fact address two quite dissimilar mechanisms: Propagule pressure determines how quickly a minimum viable population can be established, whereas colonization pressure increases the chances of introducing a suitable species. Our experiment does not completely disentangle these two aspects. However, the mean number of species introduced per run was very similar across scenarios (66.4 species for a propagule pressure of 1, 63.5 species for a propagule pressure of 10). Therefore, the difference in colonization pressure between scenarios was small, and the difference in invasion rates can be primarily attributed to propagule pressure sensu stricto.

### 4.2 Temperature and disturbance

The interaction between temperature (our surrogate for productivity) and disturbance was not as straight‐forward as we had expected. The dynamic equilibrium model (Driscoll, 2017; Huston, 2004) predicts that invasibility approximately increases with native diversity, which is high when both productivity and disturbance are high, or when both are low. This is because high productivity with low disturbance leads to population extinction through competitive exclusion, while low productivity with high disturbance means small populations that go extinct stochastically. Indeed, as described by Huston (2004), we did observe a clear peak of mean native species richness in the low‐temperature, low‐disturbance scenarios (Figure S2). Despite this, maximum invasibility did not coincide with either low‐temperature/low‐disturbance or high‐temperature/high‐disturbance scenarios, but was driven almost entirely by temperature (Figure 3). This apparent mismatch of our model with theory may raise some questions at first, but can be explained by a closer look at disturbance and its influence on invasion processes.

The interplay between productivity and disturbance and its effect on species richness and composition has long been discussed in the theoretical literature (e.g., Catford et al., 2012; Chesson, 2000) and shown in multiple empirical studies (e.g., Huebner et al., 2018; O'Connor et al., 2017). However, these interactions are subject to several preconditions. Firstly, Buckley et al. (2007) point out that disturbance may increase the invasibility of a habitat, but can also be a cause of mortality for alien species, reducing invasibility again. Thus, if disturbance affects natives and aliens equally, these two contrary effects may cancel each other out, leaving no net change. This seems to be the case in our model, as disturbance‐driven mortality was species‐agnostic. Secondly, the effects of disturbance on native and alien communities are likely to change over the full gradient of disturbance intensity (Catford et al., 2012). Analyzing that many disturbance levels was beyond the scope of this study, however. Therefore, our experiment only reflects two points on this gradient and may thus give an incomplete picture. And thirdly, an invaded community's response to disturbance can be strongly modulated by the trait composition of its native and alien species (Kempel et al., 2013; Mata et al., 2013). This includes traits that are represented in our model, which will be discussed in the next section.

### 4.3 Traits of invasive species

Although sometimes questioned in studies on species invasions (cf. Catford et al., 2009, but see Thuiller et al., 2006), our results provide some insights into an “invasion syndrome” relative to the native community (Novoa et al., 2020). In fact, invasive species are more similar to native species than alien species in terms of their trait characteristics (cf. Küster et al., 2010). This suggests that invasive species have to pass similar environmental filters to natives in order to successfully establish in a new location.

Despite the above‐mentioned similarities between invasive and native species, invasive species still showed key differences. In fact, we observe here a trait‐specific adjustment of generalism and specialism: Invasives have a higher precipitation tolerance than natives, but a lower temperature tolerance. We suggest that this phenomenon is driven by the dual requirements of establishment and landscape spread. In order to establish in an already settled habitat, invasive species require a high degree of adaptation, as well as sufficient propagule pressure, in order to avoid competitive exclusion by native species (Alzate et al., 2020; Kempel et al., 2013). On the other hand, generalism (coupled with strong dispersal abilities) is beneficial for landscape spread (Irl et al., 2021). In the context of our model, the temperature specialization seen in invasives is a key determinant of their high adaptation values (Figure 5d,g), giving them a competitive advantage at their point of entry on the island. This is aided by their larger adult biomass (Figure 5e), which decreases their density‐independent mortality rates yet further due to the MTE. At the same time, their high precipitation tolerance gives them a wide potential habitat range, allowing them to spread across the lowland, as area decreases with temperature via changes in elevation (Figures 1 and 5c). As expected, invasive species also show large dispersal distances (Figure 5a,b). The difference in trait characteristics between species categories might also indicate different evolutionary backgrounds. In this respect, our island communities are the result of several hundreds of years of ecological interactions between species and the environment. This is especially evidenced by their decreased dispersal ability, which is a typical island adaptation (Burns, 2019). They are, therefore, sensitive toward invasive species that (1) were not restricted by those interactions and (2) exhibit trait syndromes different from those represented in the community. Additionally, invasive species are bigger than natives, but not as big as (unsuccessful) alien species (Figure 5e). Thus, the invasion syndrome features adaptation to similar conditions as the invaded native community, but sufficiently different other key traits (e.g., precipitation tolerance), unbounded by the local evolutionary history, to fill areas of the trait space with the least overlap with native species. If this overlap is nonetheless enough to induce competitive exclusion, superior competitive abilities allow invasive species to outperform, and potentially replace, native species (cf. Flory & Clay, 2010; Pyšek et al., 2012). Indeed, we could observe such invasion‐driven extinctions in a few of our simulations.

## 5 FUTURE CONSIDERATIONS

An important result that warrants further investigation in future studies is the trait‐specific trade‐off between generalism and specialism. The impact of traits on invasion success has been shown to be strongly context‐dependent, also with regard to generalism (Fristoe et al., 2021). Our observed decrease in temperature tolerance, but increase in precipitation tolerance, is likely contingent upon the landscape configuration of our simulated island, which causes particular temperature values to have larger areas. It is known that points of entry and landscape structure are important contributors to invasion patterns (Catford et al., 2011). Whereas our result already provides a mechanistic explanation of how different landscape configurations may affect invasion success via niche‐based processes, it would be interesting to investigate in detail how the spatial factors of entry point as well as of different environmental combinations and regions affect trait trade‐offs, and subsequently, the type of species that are likely to invade.

Although beyond of the scope of this study, the current model allows us to consider genomic traits in the characterization of species. In a previous study, Leidinger et al. (2021) show how environmental variation interacted with the number of genes and genomic variation of communities. Similarly, invasive species might display particular genomic profiles which enable them to quickly adapt to novel environments, for example, by high standing variation or phenotypic plasticity (Zenni et al., 2014). To increase computational feasibility and preclude possible confounding effects, we chose not to include these effects in the present study. However, we expect future experimental designs to account for variation in genomic traits and to allow for mutations and thus for rapid evolution when investigating species invasions.

## 6 CONCLUSION

Here, we investigated the relative importance of propagule pressure, productivity, disturbance, and the difference in traits between native and invasive species for the success of species invasions. This broad experimental and analytical setup demonstrates the utility of individual‐based mechanistic models for understanding biological invasions. Our results hold relevance for policy and management, as they reinforce the importance of reducing the import of alien species. This is true of alien species in general, but even more so for those showing high dispersal abilities coupled with generalism for key environmental properties, as well as those coming from habitats with similar conditions as native ecosystems.

## CONFLICT OF INTEREST

The authors declare no conflict of interest.

## AUTHOR CONTRIBUTIONS

**Daniel Vedder:** Conceptualization (equal); Formal analysis (lead); Investigation (lead); Methodology (equal); Software (supporting); Visualization (lead); Writing‐original draft (lead); Writing‐review & editing (equal). **Ludwig Leidinger:** Formal analysis (supporting); Methodology (equal); Software (lead); Supervision (supporting); Visualization (supporting); Writing‐review & editing (supporting). **Juliano Sarmento Cabral:** Conceptualization (equal); Methodology (equal); Project administration (lead); Resources (lead); Supervision (lead); Writing‐review & editing (equal).

## Supporting information

Appendix S1

Appendix S2

## Data Availability

All data are reproducible using the simulation source code archived at https://doi.org/10.5281/zenodo.5602906. The development version may be found at https://github.com/CCTB‐Ecomods/gemm.
